# Cancer as a Hidden Catalyst: Rethinking Postoperative Atrial Fibrillation After Cardiac Surgery

**DOI:** 10.3390/jcm15124456

**Published:** 2026-06-09

**Authors:** Sotiris Kyriakou, Marios Markantonis, Panos Georghiou, Filippos Triposkiadis, Amalia Georgiou, Konstantinos Lampropoulos, Argyris Kyriakou, Nikolas Iosif, Georgios P. Georghiou

**Affiliations:** 1 Barts and The London School of Medicine and Dentistry, Queen Mary University of London, London E1 2AD, UK; s.kyriakou@nhs.net (S.K.); panos.georghiou@gmail.com (P.G.); 2 School of Medicine, European University Cyprus, 2404 Nicosia, Cyprus; mm202135@students.euc.ac.cy (M.M.); ftriposkiadis@gmail.com (F.T.); k.lampropoulos@euc.ac.cy (K.L.); 3 Department of Cardiology, Marien Hospital Düsseldorf, 40479 Düsseldorf, Germany; amalia.georgiou.med@gmail.com; 4 Barking, Havering and Redbridge University Hospitals NHS Trust, London RM1 2BA, UK; argyris.kyriakou6@nhs.net; 5 Faculty of Medicine & Surgery, University of Turin, 10126 Turin, Italy; nikolas.iosif.md@gmail.com; 6 Department of Surgery, Gray Faculty of Medical & Health Sciences, Tel Aviv University, Tel Aviv 6997801, Israel

**Keywords:** postoperative atrial fibrillation, cardiac surgery, malignancy, cardio-oncology, cancer therapy-related cardiotoxicity, perioperative risk stratification, atrial vulnerability, inflammation

## Abstract

Postoperative atrial fibrillation (POAF) is the most frequent post-cardiac surgical arrhythmia and is associated with haemodynamic instability, longer hospital stays, higher healthcare utilisation, stroke and adverse long-term outcomes. Conventional models of POAF focus on advancing age, pre-existing atrial substrate, increasing operative complexity, inflammatory responses related to cardiopulmonary bypass, disturbances in autonomic balance, and acute metabolic insults during the perioperative period. Although these explanations are important, they may be incomplete. Malignancy and the cancer-associated systemic environment involve biological processes potentially relevant to postoperative atrial vulnerability, such as chronic inflammation, oxidative stress, impaired vascular function, hypercoagulability, altered immune response, frailty, and treatment-related myocardial, pericardial, or conduction-system injury. This review assesses whether malignancy should be regarded as an underrecognised modifier of susceptibility rather than as a coincidental comorbidity. Most available data are extrapolated from cardio-oncology and thoracic oncology studies or general studies related to atrial fibrillation (AF) rather than studies focused on cardiac surgery. While malignancy cannot be dismissed as irrelevant to the generation of POAF, it also does not permit causal inference. Future studies should move beyond binary cancer status and incorporate cancer activity, treatment exposures, biomarker profiles, and atrial substrate measures to determine whether malignancy improves perioperative POAF risk stratification.

## 1. Introduction

Despite reductions in the incidence of postoperative atrial fibrillation (POAF) reported in some populations following the adoption of “off-pump” techniques and strategies that reduce direct atrial stimulation, POAF remains an important source of morbidity after cardiac surgery. The reported frequency of POAF varies according to procedure type, rhythm-monitoring intensity, and case definition. Contemporary observational studies and systematic reviews report high rates of POAF after coronary artery bypass grafting, valve repair or replacement, and combined cardiac procedures [1,2,3,4,5]. POAF is associated with haemodynamic instability, prolonged intensive care and hospital stay, greater healthcare resource utilisation, and increased risk of subsequent adverse cardiovascular events, including recurrent atrial fibrillation (AF) and ischaemic stroke. POAF should therefore be regarded as a clinically significant postoperative syndrome rather than a benign transient rhythm disturbance [1,2,3,4,5]. This review examines whether malignancy is an underappreciated modifier of susceptibility to POAF in patients undergoing cardiac surgery. Current evidence does not establish causality or support cancer-specific perioperative recommendations. Nevertheless, available data suggest that malignancy and cancer therapies may amplify established mechanisms of postoperative atrial vulnerability.

Two prior publications from our group have addressed related aspects of this topic and warrant explicit distinction. Georghiou GP et al. conducted a prospective clinical study reporting POAF incidence in relation to cancer status after cardiac surgery, providing preliminary observational data but not a mechanistic synthesis [6]. A subsequent narrative overview introduced the conceptual framework linking cancer-associated biology to perioperative atrial vulnerability [7]. The present review extends both prior works in several substantive ways. It proposes a structured mechanistic hierarchy that distinguishes substrate-based from trigger-based POAF, introduces an explicit evidence hierarchy across cancer therapy classes, and presents a candidate biomarker and phenotyping panel for future prospective studies. It also provides dedicated analysis of competing explanations, including frailty, multimorbidity, and surveillance bias. Crucially, it distinguishes between active malignancy, prior inactive disease, and definitively cured cancer, a distinction that was incompletely addressed in earlier work. Taken together, this review aims to move the conversation from observational association toward a framework that can guide future study design.

## 2. POAF as a Substrate-Trigger Syndrome After Cardiac Surgery

Current models of POAF emphasise acute perioperative stress acting on a susceptible atrium. Relevant stressors include surgical trauma, cardiopulmonary bypass, oxidative injury, catecholamine release, pericardial inflammation, atrial stretch, fluid and electrolyte disturbance, and transient organ dysfunction [8,9,10,11,12,13]. Although the acute insult may resolve soon after surgery, the substrate it reveals or exacerbates may persist. Despite progress in understanding POAF mechanisms, available prediction tools remain only modestly effective, suggesting that clinically relevant determinants of susceptibility remain incompletely captured.

Rather than representing a single postoperative event, POAF should be understood as a syndrome. For some patients, for example, POAF is primarily trigger-based and resolves as the immediate postsurgical physiological disturbances subside; however, for others, POAF reveals an underlying atrial pathology with long-term implications. This distinction is clinically relevant, as it suggests that a transient arrhythmia triggered by acute stress does not carry the same implications as an arrhythmia that reveals an underlying atrial pathology and portends recurrence, thromboembolic events, and broader cardiovascular instability [1,2,3,4,5,8].

While conventional models for predicting POAF remain clinically useful, they are biologically incomplete. These models identify age, type of operation, atrial size, prior heart disease, and standard perioperative variables as predictors. However, they account poorly for chronic inflammatory burden, treatment-induced myocardial damage, occult radiation-induced fibrosis, cancer-related endothelial dysfunction, and diminished physiological reserve as represented by frailty and sarcopenia [8,9]. If malignancy is relevant, it will likely be because current models inadequately capture how oncologic biology and treatment history influence the biological substrate on which postoperative triggers act.

## 3. Malignancy as a Systemic Modifier of Atrial Vulnerability

Malignancy may contribute to POAF susceptibility for reasons that extend beyond its higher prevalence in older surgical populations. It encompasses chronic inflammation, thrombosis, endothelial dysfunction, autonomic disruption, metabolic stress, frailty, sarcopenia, and exposure to potentially cardiotoxic therapies—all of which may interact with established mechanisms of AF development [14,15,16,17,18,19,20]. Treating cancer as a routine background comorbidity may therefore obscure important clinical context.

There is considerable human-derived evidence supporting the notion that patients who experience POAF typically enter surgery with pre-existing structural and electrophysiological abnormalities, rather than acquiring all relevant substrate postoperatively [21,22]. Concurrently, numerous studies examining perioperative mechanisms suggest that pericardial inflammatory signalling, oxidative stress, and inflammasome activation are key elements in creating the postoperative arrhythmogenic environment [23,24,25]. These findings suggest that malignancy may intensify established substrate–trigger interactions rather than introduce a separate model of POAF.

## 4. Shared Mechanistic Pathways Between Cancer and POAF

The following mechanistic links vary in their evidential strength: some, such as the role of BTK inhibitors, are supported by direct clinical data, while others, such as cancer-related inflammation as a primer for POAF, remain biologically plausible but largely theoretical. The strongest mechanistic overlap between malignancy and POAF involves inflammation. Increasingly, modern AF biology implicates inflammatory signalling in atrial fibrosis, abnormal calcium handling, conduction slowing, and substrate persistence [7,26,27]. Notably, many cancers impose chronic inflammatory burdens, rather than the episodic inflammatory surges more typical of uncomplicated perioperative stress. Consequently, when such a patient undergoes cardiac surgery, the postoperative inflammatory surge occurs on top of a primed inflammatory milieu. The relevant issue may not be the absolute magnitude of the inflammatory surge, but rather that it occurs in a host already primed toward atrial instability. However, this interpretation remains theoretical because of the lack of information regarding perioperative biomarker and atrial tissue data in cancer patients who have undergone cardiac surgery [7,26].

Another relevant pathway involves hypercoagulability and endothelial dysfunction. Although cancer-associated coagulation activation is generally discussed in relation to venous thromboembolism, thrombo-inflammation also intersects with endothelial injury, platelet activation, microvascular dysfunction, and local inflammatory propagation, all of which may be relevant to atrial substrate [7,26,27]. Cardiac surgery further perturbs these domains via bypass, transfusion, myocardial and pericardial injury, and postoperative inflammatory activation. Therefore, patients with active or recently treated cancer may enter the perioperative period already physiologically depleted, before the typical postoperative stressors become arrhythmogenic.

Frailty, cachexia, sarcopenia, and autonomic dysregulation can be conceptualised either as confounders or as mechanisms, since they are not exclusive to malignancy and may each contribute independently to AF risk. Nevertheless, they remain relevant when assessing cancer’s role in perioperative susceptibility, regardless of whether malignancy exerts its effects through these intermediaries or through more tumour-specific pathways. For perioperative assessment, the key issue is not simply whether cancer is present, but which oncological consequences the patient brings into the operating room.

The shared pathological terrain between malignancy and AF is not complete, and current evidence does not support a bidirectional disease model. However, cardiac surgery may represent the clinical context in which this convergence becomes most apparent—transforming latent arrhythmic susceptibility into overt POAF [7,26,27].

Atrial myopathy represents an additional mechanistic concept that is particularly relevant to this discussion. Defined by structural and functional alterations of the left atrium—including impaired reservoir and conduit strain, left atrial enlargement, P-wave abnormalities, and elevated NT-proBNP—atrial myopathy shares key pathophysiological cascades with both AF and cancer, including chronic inflammation, extracellular matrix remodelling, oxidative stress, and fibrosis [28]. Importantly, markers of left atrial (LA) myopathy have been shown to carry prognostic value for adverse outcomes, including stroke and dementia, even in patients in sinus rhythm, suggesting that structural atrial vulnerability can exist and be quantified before AF becomes clinically overt [28]. In the perioperative context, cancer-associated chronic inflammation and treatment-related cardiac injury may accelerate the development or progression of atrial myopathy, thereby establishing the atrial substrate on which surgical triggers act. This raises an important hypothesis for future investigation: whether preoperative markers of LA myopathy—specifically LA volume index, reservoir strain, and NT-proBNP—measured in patients with malignancy who are in sinus rhythm prior to cardiac surgery, carry independent prognostic value for the development of POAF. A prospective study incorporating such measures alongside cancer activity classification and treatment exposure data would directly test this hypothesis and could refine preoperative risk stratification in this population.

**Figure 1:**
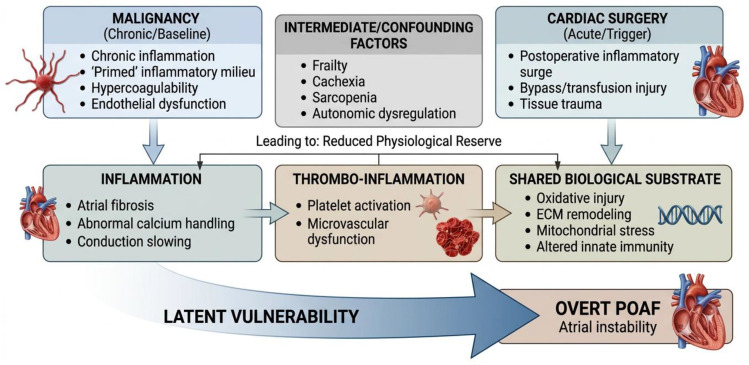
Malignancy-related substrate-trigger model of POAF. This figure represents a proposed conceptual framework illustrating how malignancy-related biological factors may converge with acute surgical stressors to promote POAF. It does not imply established causal relationships.

Figure 1 illustrates how malignancy-related vulnerability and acute cardiac-surgical stressors may converge to promote POAF.

## 5. POAF as Marker, Mediator, or Both

Although POAF may contribute to later adverse outcomes, in many patients it may also reveal pre-existing atrial disease with longer-term clinical significance [1,2,3,4,5,8,21,22]. If cancer contributes to this underlying atrial substrate, its relevance may not lie in creating a completely new postoperative phenomenon. Rather, malignancy may increase atrial susceptibility to the point where the physiological stress of surgery exposes disease processes that were already developing. This interpretation may help explain why POAF, recurrent AF, and later adverse outcomes often cluster together, without necessarily implying a simple linear causal relationship.

## 6. Cancer Therapy Exposures

The strength of evidence linking individual cancer therapy classes to POAF varies considerably, ranging from direct clinical data for BTK inhibitors to indirect mechanistic plausibility for most other agents. While a history of cancer is certainly a component of the overall burden of comorbidity among patients who undergo cardiac surgery, it should not be treated as a generic comorbidity. Many patients presenting for cardiac surgery have undergone treatments that may have altered myocardial reserve, affected the conduction system, damaged the pericardium, influenced the autonomic nervous system, modified vascular biology, or altered atrial tissue itself through chemotherapy, targeted therapies, or thoracic irradiation. However, although the evidence base remains somewhat uneven across different classes of cancer therapy, these exposures should be distinguished by mechanism, timing, and magnitude of effect [29,30,31,32,33].

Anthracyclines are particularly important because of their well-established cardiotoxic effects, although their impact on AF may occur indirectly. For example, anthracyclines can produce oxidative injury to the myocardium, impair mitochondrial function with reduced ATP production, promote myocardial fibrosis, and result in impaired left ventricular function. These structural and metabolic changes may render hearts previously exposed to anthracyclines vulnerable to ischaemia–reperfusion injury, catecholamine surges, and mechanical stress during cardiac surgery, and thus potentially more susceptible to POAF [34].

Bruton tyrosine kinase (BTK) inhibitors represent the most direct therapy class for which a demonstrable link with increased risk of POAF has been reported. Specifically, BTK inhibitors, especially ibrutinib, have been associated with pro-arrhythmic effects through multiple lines of evidence [35,36]. This distinction is important because it suggests that cancer therapy may increase POAF risk through mechanisms more specific than a generalised comorbidity burden. However, this observation should not be extrapolated broadly across all classes of cancer therapy. Rather, it is more appropriate to conclude that patients who are taking BTK inhibitors, or who have been exposed to them recently, and are undergoing major cardiac interventions warrant particular attention.

In contrast, there is greater heterogeneity in the relationships between other classes of cancer therapy (e.g., immune checkpoint inhibitors, fluoropyrimidines, human epidermal growth factor receptor 2 (HER2)-directed therapies, androgen deprivation therapy) and AF [34,35,36,37,38,39]. For example, immune checkpoint inhibitors have been associated with myocarditis, pericarditis, conduction abnormalities, and immune activation; fluoropyrimidines are more commonly linked to ischaemia and vasospasm than to specific AF risk; HER2-directed therapies may influence POAF risk through effects on myocardial reserve; and androgen deprivation therapy may modulate arrhythmic risk through cardiometabolic and vascular pathways [37,38,39]. Thoracic radiotherapy may be the most anatomically compelling long-term exposure because of its potential effects on the atria, sinoatrial node, pericardium, autonomic ganglia, and mediastinum, and substructure-based analyses have linked radiation dose to AF risk [40,41]. The key point is not that all these exposures are equivalent, but that perioperative cardiovascular assessment should distinguish between them rather than combining heterogeneous treatment histories to a single aggregate variable [40,41].

Table 1 summarises the major anticancer therapy classes with potential relevance to perioperative atrial vulnerability and POAF susceptibility.

Evidence categories reflect the consistency, directness, and volume of published data in relation to POAF after cardiac surgery specifically. High: consistent signal from multiple prospective or randomised studies with a direct AF or POAF endpoint. Moderate: supportive observational or mechanistic data, without prospective cardiac surgical evidence. Low-moderate: indirect, limited, or heterogeneous data with biological plausibility but no direct POAF signal. Low: predominantly indirect evidence via cardiometabolic or vascular pathways with no established atrial-specific mechanism.

## 7. Perioperative Stressors in the Cancer-Exposed Patient

The biological conditions created by cardiac surgery provide the backdrop against which POAF develops. Cardiopulmonary bypass activates inflammatory and coagulation cascades. Myocardial reperfusion adds oxidative insult, and local inflammation arises from atrial manipulation and pericardial blood retention. Catecholamine surge, volume shifts, and electrolyte imbalance complete the perioperative arrhythmogenic profile [23,24,25]. In this setting, cancer is best viewed as an additive stressor that may increase vulnerability to established perioperative triggers.

This interpretation aligns with what is known about selected groups of patients undergoing cardiac surgery who have either active or prior cancer. Although postoperative mortality rates may remain within “acceptable” limits in such patients, they often exhibit chronic inflammation, endothelial dysfunction, marrow suppression, anaemia, malnutrition, renal vulnerability, and prior treatment-related cardiac injury [6,42,43,44]. Collectively, these features may make otherwise standard perioperative triggers more potent in cancer-exposed patients.

**Figure 2:**
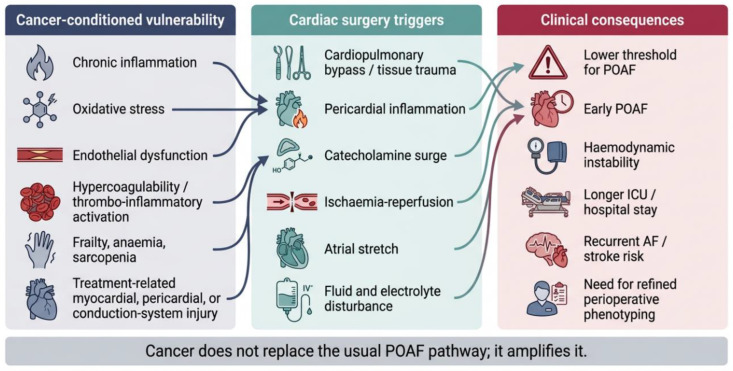
Cancer as an amplifier of POAF susceptibility. This figure is a proposed conceptual model and reflects biological plausibility rather than proven mechanistic pathways.

Figure 2 summarises how cancer-related vulnerability may amplify established cardiac-surgical triggers and lower the threshold for POAF.

## 8. Clinical Evidence

It should be noted at the outset that direct, high-quality clinical evidence specifically linking malignancy to POAF after cardiac surgery remains sparse; much of what follows is derived from registry data, indirect oncological surgery literature, and mechanistic extrapolation. There are few prospective clinical studies examining the specific relationship between cancer and POAF after cardiac surgery. Georghiou et al. provided one of the earliest prospective assessments of POAF incidence in relation to cancer status. Their findings support the possibility that malignancy and operative stress may interact in arrhythmogenic ways; however, the evidence remains preliminary and does not establish independence from operative stress, comorbidity burden, or baseline atrial vulnerability [6].

Beyond the Georghiou et al. study, much of the literature linking cancer and POAF has relied on registry data showing that cancer is increasingly prevalent among cardiac surgical populations. While these registries suggest that prior cancer exposure may influence long-term postoperative outcomes [6,42,43,44], none were designed specifically to examine cancer-specific mechanisms of POAF [6,42,43,44].

Data from non-cardiac cancer surgery suggest that POAF is neither rare nor trivial in patients undergoing cancer-related operations [45,46,47], while recurrent AF episodes after discharge may be underrecognised [45,46]. Within this broader non-cardiac oncological surgery literature, thoracic oncology represents a field with partially analogous pathophysiology. Lung resection, for example, is an area in which POAF is common and in which malignancy may exert local effects on the pulmonary veins and pericardial structures [47,48]. Taken together, these data are supportive of the hypothesis but remain indirect and cannot determine whether cancer independently increases POAF after cardiac surgery [46].

Table 2 summarises the principal published studies directly or indirectly examining POAF in cancer-exposed surgical populations.

## 9. Methodological Limitations of the Current Evidence Base

Several limitations in the current literature should shape interpretation and they include (1) crude definitions of cancer exposure, (2) incomplete documentation of both disease activity and disease stage at diagnosis, (3) inadequate detail regarding prior treatment exposures, (4) significant variability among institutions and providers regarding their use of rhythm monitoring and (5) lack of distinction between pre-existing AF and truly de novo AF that occurred after surgery. Survivorship bias may also complicate direct comparison between patients with active malignancy and those with prior cancer. Patients with very advanced malignancies are frequently unable to undergo evaluation for cardiac surgery. Thus, while cohorts of patients with a history of cancer may provide some insight into potential risks associated with undergoing cardiac surgery, they are unlikely to accurately represent the risk experienced by cohorts of patients who have active systemic malignancy, unless future studies clearly distinguish these states.

## 10. Toward More Informative Risk Stratification

Cancer is likely to inform clinical decision-making meaningfully if it is used as something more than a simple binary variable. As such, a history of cancer typically provides little additional mechanistic insight beyond that provided by a patient’s age, comorbidities, and functional reserve. A potentially more meaningful way to stratify risk based on cancer would be to incorporate more granular variables to better characterise both the status of the patient’s cancer and the nature of their previous treatments. This requires documenting cancer activity status, tumour type, disease stage, and time since diagnosis. Treatment exposures should be recorded with enough specificity to distinguish limited historical exposure from ongoing cardiovascular injury. If incorporated into a model predicting POAF, cancer is more likely to enhance discrimination through the refinement of phenotypic characterization rather than through binary classification.

## 11. Candidate Biomarkers and Phenotypic Markers

Variables most likely to add value are those that convert cancer exposure into a quantifiable cardiovascular phenotype. Candidate variables that fit this concept and have mechanistic rationale for relevance to POAF include left atrial size and deformation, natriuretic peptide levels, frailty indices, inflammatory marker levels, and selected thrombophilic or inflammatory markers [49,50,51,52,53]. This is because these variables capture physiological manifestations of malignant disease and treatment history, rather than merely documenting a history of malignancy; therefore, they may offer greater insight than a simple cancer history in patients with prior or ongoing malignancy-related exposure. As a result, prospective studies should determine whether these biomarkers and imaging measures improve discrimination, calibration, and predictive performance beyond conventional models.

Table 3 outlines candidate biomarker, imaging, and phenotypic measures that could support future prospective studies of cancer-related POAF susceptibility.

## 12. Implications for Perioperative Assessment and Management

Current evidence is insufficient to justify cancer-specific prophylactic protocols for POAF. However, it supports a more structured preoperative assessment that captures cancer activity, current and previous treatment exposures, prior thoracic radiotherapy, frailty, anaemia, thrombocytopenia, renal dysfunction, and previous cardiotoxicity [16,17,18,19,20]. Cancer history should therefore inform perioperative risk assessment as a clinically meaningful domain, rather than be recorded as a simple binary variable.

In selected higher-risk cancer-exposed patients, individualised postoperative rhythm surveillance may represent a reasonable clinical consideration—particularly when POAF would complicate anticoagulation decisions or future interventions—though this approach is not currently supported by direct trial evidence and should not be interpreted as a practice recommendation. Because cancer may increase both thrombotic and bleeding risk through thrombocytopenia, mucosal injury, drug–drug interactions, and planned invasive procedures, anticoagulation decisions should be individualised rather than determined by cancer status alone [53,54,55,56,57,58,59].

## 13. Limitations and Alternative Explanations

The principal competing explanation is that cancer is not an independent determinant of POAF, but a marker of older age, multimorbidity, frailty, anaemia, renal dysfunction, and treatment burden [54,55,56,57,58,59]. If the association attenuates after adjustment for established predictors, the interpretation would shift from tumour-related arrhythmogenicity to clustering of vulnerability.

Frailty deserves particular attention as a standalone competing explanation. Frailty is an independent predictor of POAF after cardiac surgery and is simultaneously over-represented in cancer populations [52]. If the association between malignancy and POAF attenuates substantially after adjustment for validated frailty indices, this would suggest that cancer’s apparent effect is mediated principally through reduced physiological reserve rather than tumour-specific biology. Current observational studies have not performed this adjustment with sufficient granularity to settle this question.

Multimorbidity represents a closely related alternative. Patients with a cancer history disproportionately accumulate hypertension, diabetes, renal dysfunction, and anaemia—each of which independently predicts POAF and atrial remodelling. If these comorbidities are incompletely captured or adjusted for, the residual cancer effect may reflect comorbidity clustering rather than oncologic biology per se. Future registry-based and prospective studies should apply comorbidity burden scores alongside cancer classification to disentangle these contributions.

Treatment burden, including polypharmacy, prior hospitalisations, nutritional depletion, and deconditioning from chemotherapy or prolonged illness, may also contribute to perioperative vulnerability independently of the biological effects of cancer itself. Patients with treatment-heavy histories may have impaired autonomic regulation, reduced functional capacity, and diminished cardiopulmonary reserve that are not captured by standard preoperative risk scores and that would lower the threshold for postoperative arrhythmia regardless of the tumour’s direct biological influence. Adequately accounting for this dimension will require more granular treatment history documentation than is currently standard in cardiac surgical databases.

Reverse causality and surveillance bias add further uncertainty. Observational studies have reported increased detection of occult cancer after new-onset AF, particularly within the first year, but this likely reflects intensified medical surveillance and shared risk architecture rather than a simple causal pathway [58,59,60]. Mendelian randomization studies add further caution by failing to show substantial shared genetic predisposition across cancer subtypes [61,62]. These observations do not exclude a cancer–POAF relationship after cardiac surgery, but they do argue against treating association as mechanism [59].

**Figure 3:**
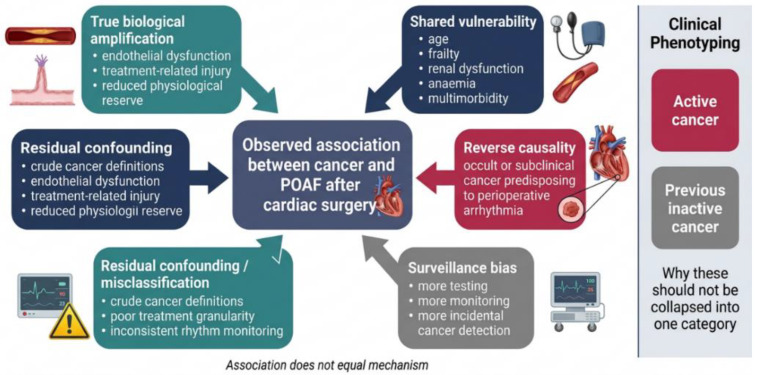
Competing explanations for cancer–POAF associations. This figure summarises alternative interpretations of the observed association and is intended to highlight the need for refined clinical phenotyping rather than to imply definitive causal conclusions.

Figure 3 summarises competing explanations for the observed association between cancer and POAF, highlighting the need for refined clinical phenotyping.

## 14. Distinguishing Active from Prior Malignancy

A critical distinction must be made between individuals with active metastatic disease and/or recent systemic therapy for their cancer and those with previously treated and now definitively cured cancer. Many databases currently collapse these two categories together. Such practices tend to diminish the gradient of biological relevance that this area of investigation seeks to understand. Future studies must distinguish cancer activity status (e.g., active, inactive), stage (e.g., localised, regional) and treatment timing (e.g., past year vs. years ago) to effectively assess the biological impact of cancer on POAF.

## 15. Overall Interpretation of the Evidence

Taken together, the available evidence supports malignancy as a plausible modifier of POAF susceptibility, but not yet as an independently validated determinant of perioperative risk. The main unresolved issue is whether cancer-related biology adds predictive value beyond the comorbidities and frailty with which it commonly clusters. The most appropriate way to investigate this question is within existing POAF frameworks. Current data suggest that cancer and its treatments may augment inflammation, oxidative stress, endothelial dysfunction, immune activation, and reserve depletion, thereby intensifying mechanisms already known to promote AF after surgery. Future studies should determine whether malignancy acts chiefly as a chronic biological preconditioner of the atrium, a marker of diminished systemic resilience, or both.

## 16. Future Directions

Progress in this field will depend on prospective phenotyping rather than retrospective association alone. A useful study design would enrol consecutive adult patients undergoing cardiac surgery and classify them as having no cancer history, prior inactive cancer, or active cancer. Tumour type, stage, time since diagnosis, prior thoracic radiotherapy, cumulative anthracycline exposure, recent systemic therapies, and previous cardiotoxicity should be captured prospectively rather than inferred from administrative data. Continuous in-hospital rhythm monitoring combined with structured post-discharge surveillance would help distinguish transient POAF from recurrent AF. Analyses should incorporate competing-risk methods for non-cardiovascular mortality, treat cancer therapies as time-varying exposures where appropriate, and test whether cancer-related variables improve discrimination, calibration, and reclassification beyond existing models [48,53,63].

## 17. Conclusions

Malignancy should not yet be regarded as an independently validated determinant of POAF after cardiac surgery, but it should not be dismissed as a passive background comorbidity. Current evidence supports a biologically plausible framework in which cancer activity, treatment-related cardiotoxicity, systemic inflammation, endothelial dysfunction, hypercoagulability, frailty, and reduced physiological reserve may converge with established perioperative triggers to increase susceptibility to postoperative arrhythmia. However, the evidence remains insufficient to support causal inference, cancer-specific prophylactic protocols, or automated treatment decisions.

The immediate clinical value of this framework lies in reframing cancer history as a clinically informative risk domain rather than a binary variable. Future prospective studies should distinguish active from prior malignancy, capture treatment timing and cardiotoxic exposures, and test whether biomarkers, frailty measures, and atrial structural markers improve POAF prediction beyond existing models. Until such data is available, malignancy is best understood as a potential vulnerability state that may lower the threshold for POAF by amplifying established perioperative mechanisms, rather than as a distinct arrhythmogenic pathway.

## Figures and Tables

**Table 1:** Anticancer therapies and POAF susceptibility.

Therapy Class	Key Cardiovascular/Atrial Effects	Relevance to POAF After Cardiac Surgery	Evidence
Anthracyclines	Oxidative injury, fibrosis, left ventricular (LV) dysfunction	Indirect: reduced myocardial reserve and structural vulnerability	Moderate
BTK inhibitors	Direct atrial pro-arrhythmia, hypertension, bleeding issues	High: strongest therapy-specific AF signal, especially if recent or ongoing	High
Immune checkpoint inhibitors	Myocarditis, pericarditis, conduction disease, immune activation	Moderate: more relevant if prior cardiotoxicity or persistent inflammation	Moderate
Fluoropyrimidines	Vasospasm, ischaemia, arrhythmias, haemodynamic stress	Modest/indirect: broader cardiotoxic stress rather than specific atrial effect	Low-moderate
HER2-targeted therapies	Ventricular dysfunction, reduced cardiac reserve	Indirect: mainly relevant when reserve is impaired	Low-moderate
Androgen deprivation/AR pathway inhibitors	Metabolic dysfunction, hypertension, vascular risk, QT effects	Indirect: mainly via cardiometabolic burden	Low
Thoracic radiotherapy	Atrial/sinoatrial (SA) node fibrosis, pericardial and conduction injury, microvascular damage	Potentially high in selected patients, especially with mediastinal exposure	Moderate

**Table 2:** Published clinical studies examining POAF in cancer patients undergoing cardiac or oncological surgery.

Study	Design	Cancer Definition	POAF Incidence/Key Finding	Limitations
Georghiou et al. [6]	Prospective observational; cardiac surgery	Active or prior cancer vs. no cancer	Cancer identified as a major determinant of POAF after cardiac surgery	Single-centre; no granular treatment exposure data; does not separate active from prior cancer
Mennander et al. [42]	Registry-based (SWEDE-HEART); CABG patients	History of cancer vs. no history	Prior cancer associated with worse long-term survival post-CABG; POAF not primary endpoint	Retrospective; cancer defined as binary; no treatment detail; POAF not specifically examined
Lorusso et al. [43]	Narrative review; cardiac surgery in malignancy	Active or remitted malignancy	Cardiac surgery feasible in selected cancer patients; increased short-term morbidity reported	Review methodology; heterogeneous populations; POAF not systematically reported across studies
Chan et al. [44]	Retrospective series; cardiac surgery	History of any malignancy	Comparable early postoperative mortality; long-term outcomes poorer	Retrospective; small sample; no granular POAF data; binary cancer classification
Higuchi et al. [45]	Prospective observational; non-cardiac cancer surgery	Active malignancy requiring surgery	POAF incidence 5.4%; associated with complications and longer stay	Non-cardiac surgery; cancer itself is the surgical indication; not directly applicable to cardiac surgical context
Higuchi et al. [46]	Prospective; non-cardiac cancer surgery	Active malignancy	One-year AF recurrence rate significant in POAF subgroup; recurrence under-recognised	Non-cardiac surgery; no comparison to non-cancer patients; limited rhythm monitoring post-discharge
Inoue et al. [47]	Systematic review and meta-analysis; cancer surgery	Cancer patients undergoing resection	POAF associated with increased in-hospital mortality and complications across cancer surgery types	Predominantly non-cardiac surgical populations; heterogeneous definitions; cardiac surgery underrepresented

**Table 3:** Proposed perioperative phenotyping panel.

Domain	Candidate Measure	Rationale	Suggested Timing
Atrial structure and function	Left atrial volume index; reservoir, conduit, and contractile strain	Defines latent atrial remodelling more directly than comorbidity burden	Preoperative echocardiography
Ventricular/haemodynamic stress	B-type natriuretic peptide (BNP) or N-terminal pro-B-type natriuretic peptide (NT-proBNP)	Reflects wall stress, filling pressure, and reduced myocardial reserve	Preoperative; postoperative day 1
Myocardial injury	High-sensitivity troponin	Helps separate perioperative injury from pre-existing reserve limitation	Preoperative baseline; early postoperative
Systemic inflammation	C-reactive protein; interleukin-6; neutrophil-based indices	Quantifies inflammatory burden relevant to both cancer and POAF	Preoperative; intensive care unit (ICU) arrival; postoperative day 1–2
Thrombo-inflammatory state	Fibrinogen; D-dimer; platelet count/derived ratios	Captures coagulation activation and perioperative haemostatic disturbance	Preoperative; postoperative day 1
Frailty/nutritional status	Clinical frailty scale; gait-based or standardised frailty score; albumin; sarcopenia surrogate	Operationalises reserve depletion that may mediate part of the cancer signal	Preoperative
Local cardiac inflammatory milieu	Selected pericardial fluid cytokines or myeloperoxidase	May identify a distinct pericardial inflammatory signature in cancer-exposed patients	Intraoperative or immediate postoperative research sampling

## Data Availability

No new data were created or analysed in this study. Data sharing is not applicable to this article.

## Abbreviations

The following abbreviations are used in this manuscript:

AF	Atrial fibrillation
POAF	Post-operative atrial fibrillation
SA	Sinoatrial
BNP	B-type natriuretic peptide
NT-proBNP	N-terminal pro-B-type natriuretic peptide
BTK	Bruton tyrosine kinase
HER2	Human epidermal growth factor receptor 2
ICU	Intensive care unit
LV	Left ventricular
